# Degree of Glutathione Deficiency and Redox Imbalance Depend on Subtype of Mitochondrial Disease and Clinical Status

**DOI:** 10.1371/journal.pone.0100001

**Published:** 2014-06-18

**Authors:** Gregory M. Enns, Tereza Moore, Anthony Le, Kondala Atkuri, Monisha K. Shah, Kristina Cusmano-Ozog, Anna-Kaisa Niemi, Tina M. Cowan

**Affiliations:** 1 Department of Pediatrics, Division of Medical Genetics, Lucile Packard Children’s Hospital, Stanford University, Stanford, California, United States of America; 2 Department of Pathology, Stanford University, Stanford, California, United States of America; 3 Department of Genetics, Stanford University, Stanford, California, United States of America; RIKEN Advanced Science Institute, Japan

## Abstract

Mitochondrial disorders are associated with decreased energy production and redox imbalance. Glutathione plays a central role in redox signaling and protecting cells from oxidative damage. In order to understand the consequences of mitochondrial dysfunction on *in vivo* redox status, and to determine how this varies by mitochondrial disease subtype and clinical severity, we used a sensitive tandem mass spectrometry assay to precisely quantify whole blood reduced (GSH) and oxidized (GSSG) glutathione levels in a large cohort of mitochondrial disorder patients. Glutathione redox potential was calculated using the Nernst equation. Compared to healthy controls (n = 59), mitochondrial disease patients (n = 58) as a group showed significant redox imbalance (redox potential −251 mV±9.7, p<0.0001) with an increased level of oxidation by ∼9 mV compared to controls (−260 mV±6.4). Underlying this abnormality were significantly lower whole blood GSH levels (p = 0.0008) and GSH/GSSG ratio (p = 0.0002), and significantly higher GSSG levels (p<0.0001) in mitochondrial disease patients compared to controls. Redox potential was significantly more oxidized in all mitochondrial disease subgroups including Leigh syndrome (n = 15), electron transport chain abnormalities (n = 10), mitochondrial encephalomyopathy, lactic acidosis and stroke-like episodes (n = 8), mtDNA deletion syndrome (n = 7), mtDNA depletion syndrome (n = 7), and miscellaneous other mitochondrial disorders (n = 11). Patients hospitalized in metabolic crisis (n = 7) showed the greatest degree of redox imbalance at −242 mV±7. Peripheral whole blood GSH and GSSG levels are promising biomarkers of mitochondrial dysfunction, and may give insights into the contribution of oxidative stress to the pathophysiology of the various mitochondrial disorders. In particular, evaluation of redox potential may be useful in monitoring of clinical status or response to redox-modulating therapies in clinical trials.

## Introduction

Inherited disorders of the mitochondrial respiratory chain can affect any organ system and are associated with significant morbidity and mortality [1]. These disorders are caused by a wide array of mutations in either the mitochondrial or nuclear genome, and encompass a broad range of molecular, biochemical and phenotypic features [1]. Collectively, they are relatively common compared with other inborn errors of metabolism, with an estimated prevalence of at least 1 in 5,000 individuals [2]. Despite this high prevalence, there are limited therapeutic options for patients; only a few controlled clinical trials have been performed and there are no FDA-approved therapies designed specifically for mitochondrial disorders [3], [4]. This may in part be due to our incomplete understanding of disease pathophysiology, including possible differences in pathogenic mechanisms between the disease subtypes, as well as a paucity of reliable biomarkers with which to monitor disease progression and response to therapy [5].

Mitochondrial dysfunction leads to decreased energy production and increased production of free radicals including reactive oxygen and nitrogen species (RONS) [6]–[10], particularly by respiratory chain complexes I and III [11]–[14]. Although RONS are important products of the mitochondrial electron transport chain (ETC), serving as intracellular messengers and signals for a variety of cellular functions including mitochondrial biogenesis and apoptosis [15], abnormal increases in RONS production result in damage to cellular lipids, proteins and nucleic acids as intracellular antioxidant systems become overwhelmed [13], [16].

Glutathione (L-γ-glutamyl-L-cysteinylglycine) is the most abundant intracellular thiol tripeptide and is present in all mammalian tissues, and plays a key role in cellular defense against oxidant damage [17]. Normal tissue levels range from approximately 0.1 mM to 10 mM, with the highest concentrations reported in liver, spleen, kidney, lens, erythrocytes and leukocytes [18]. Blood GSH levels have been considered to reflect the overall body GSH status, and hence are a potential indication of disease risk in humans [18]–[22]. Low glutathione concentrations are present in muscle samples obtained from individuals with primary mitochondrial disease [23]. Glutathione deficiency has also been reported in a variety of disorders associated with impaired mitochondrial function, including Friedreich ataxia [20], Leigh syndrome [21], organic acidemias [22], [24], and neurological disorders such as Alzheimer disease [25], Parkinson disease [26] and amyotropic lateral sclerosis [27]. These studies support the idea that glutathione levels may be a useful indicator of overall redox balance, and that this metric may give insights into various types of mitochondrial dysfunction.

Using a high-dimensional fluorescence-activated cell sorter (Hi-D FACS) technique, we previously demonstrated decreased intracellular glutathione (iGSH) concentration in CD4 and CD8 lymphocytes derived from individuals with primary or secondary mitochondrial dysfunction [28]. To extend these findings, we recently reported a new liquid chromatography-tandem mass spectrometry (LC-MS/MS) method for the accurate and sensitive quantitation of reduced (GSH) and oxidized (GSSG) glutathione levels in whole blood [29]. Here we utilize this approach for the evaluation of glutathione redox status in a large cohort of patients with mitochondrial disease. In order to understand more clearly the *in vivo* consequences of ETC dysfunction, we now report the results of LC-MS/MS analysis of GSH and GSSG concentrations and redox potential in whole blood from individuals with different subtypes of primary mitochondrial disorders. We were particularly interested in determining 1) if such measurements hold promise as biomarkers in mitochondrial disorders as a whole; 2) if redox imbalance varies depending on the subtype of mitochondrial disease; and 3) if redox imbalance is a sensitive indicator of clinical status.

## Materials and Methods

### Subject Population

Blood samples (n = 87) from individuals with mitochondrial disease (n = 58) were obtained during routine clinic visits during periods of relative health (Tables S1 to S6). Seven additional samples were obtained from patients during hospital admission for management of metabolic crises (subjects 28, 37, 53, 59, 60, 61, and 62), including four patients not otherwise tested (Table S7). The total study population therefore consisted of 94 blood samples from 62 individuals. Only subjects with “definite” mitochondrial disease as defined by the modified Walker criteria [30] were studied, and included patients with Leigh syndrome (n = 15), electron transport chain (ETC) abnormalities (n = 10), mitochondrial encephalomyopathy, lactic acidosis and stroke-like episodes (MELAS) (n = 8), mtDNA deletion syndrome (n = 7), mtDNA depletion syndrome (n = 7), and miscellaneous other mitochondrial disorders (n = 11). The mtDNA deletion patients all carried a large mtDNA deletion and had either a Kearns-Sayre syndrome (n = 4) or Pearson syndrome (n = 3) phenotype. The Pearson syndrome patients did not have clinically significant anemia at the time of blood sampling (Table S4). “Metabolic crisis” was defined as worsening clinical status associated with the need for hospitalization for management. Metabolic abnormalities, such as metabolic acidosis, lactic acidosis, and abnormalities including increasing blood transaminase, creatine kinase, or glucose levels were associated with the episodes (Table S7). The Stanford University Institutional Review Board (IRB) approved this study, and samples were collected only after obtaining written informed consent from participants or from the parents or legally authorized representatives of children or minors enrolled in this study. Whole blood GSH and GSSG concentrations from control subjects (residual blood samples from 59 healthy individuals [31 male, 28 female; age 1–87 years, mean 25 years]) have previously been published [29], and were used for comparisons in this study.

### Assessment of Clinical and Antioxidant Status

Clinical status was determined using the Newcastle Paediatric Mitochondrial Disease Scale (NPMDS) [31], a measure comprised of objective assessments of current function, system-specific involvement and current clinical (sections I-III) as well as a quality of life questionnaire (section IV). NPMDS scores were determined for 23 patients during outpatient clinic visits and three patients during metabolic crises, and are presented as the sum of scores for sections I-III, the score for section IV, and the overall sum (Tables S1 to S7). In addition, three adults had clinical status determined using the Newcastle Mitochondrial Disease Adult Scale (NMDAS) (Tables S2, S3 and S6). Information regarding pharmacologic supplementation with antioxidants, including N-acetylcysteine, ascorbate, vitamin E, α-lipoic acid or coenzyme Q_10_, was obtained by patient or parent report at the time of sample collection. Antioxidant supplementation was not standardized among patients.

### Glutathione Measurements

Sample collection, preparation and analysis were carried out as previously described [29]. In brief, blood samples were refrigerated immediately following collection and processed within 24 hours by adding a precipitating solution of sulfosalicylic acid containing the derivatizing agent N-ethylmaleimide (NEM). Derivatized samples were stored at −80°C prior to analysis. Samples were analyzed by LC-MS/MS using stable-isotope internal standards of GSH (GSH-^13^C,^15^N) and GSSG (GSSG-^13^C,^15^N) (AnaSpec Inc., Fremont, CA) for quantitation. GSH-NEM and GSSG ions and fragments monitored in the positive mode using transitions *m/z* 433>304 and *m/z* 613>355, respectively. Stable-isotope internal standards were monitored as *m/z* 435>306 (GSH-^13^C, ^15^N-NEM) and *m/z* 617>359 (GSSG-^13^C,^15^N). Data from MS/MS were acquired with Analyst software, version 1.4 and calculations were performed with Chemoview application, version 1.2b9.

### Calculation of Redox Potential

The Nernst equation, *E_h_* (mV) = *E_0_*+30log([GSSG]/[GSH]^2^), was used to calculate whole blood redox potential [32], where GSH and GSSG are concentrations in moles/liter and *E_0_* is –264 mV at pH 7.4 [33]. The value for *E_0_* at pH 7.4 is based on an adjustment of –5.9 mV for every 0.1 increase in pH, taking *E_0_* at pH 7.0 as –240 [32], [33].

### Statistical Analysis

Based on our previous experience with the measurement of intracellular GSH in mitochondrial disease patients [28], we expected to detect GSH deficiency in the current study population compared to controls. Therefore, sample size was calculated based on a mean difference of 20%, as has been reported in previous research [28], with a power of 0.80 and an alpha (α) of 0.05. A significant difference was determined at p<α. Analysis of variance (ANOVA) was used to determine group differences in mitochondrial disease subgroup (Leigh syndrome, ETC disorders, MELAS, mtDNA deletion syndrome, mtDNA depletion syndrome, and miscellaneous) and whole blood levels of GSH, GSSG, GSH/GSSSG ratio, and redox potential. Differences between mean whole blood levels of specific disease subgroup and mean whole blood levels of controls (one-to-one comparisons) were conducted using Student’s t-test. Spearman’s correlation was used to assess the correlation between disease severity (as measured by the NPMDS sections I to III combined, section IV, and sections I to IV combined), age, and mutant load with whole blood glutathione data. Multiple blood samples were available from 18 subjects (Tables S1 to S6). In order to decrease potential bias, in cases of multiple samples a mean value for each subject was used for data analysis. For analysis of NPMDS scores, the clinical status sections of the NPMDS or NMDAS (sections I to III) were initially combined and analyzed separately from section IV (quality of life questionnaire). NPMDS sections I to IV were also combined and analyzed in aggregate. Data were analyzed using SAS software, version 9.4 (SAS Institute Inc., Cary, NC, USA).

## Results

### Subject Population and Sample Collection

Subject demographics and diagnoses are shown in Table 1. In total, 94 samples from 62 subjects with definite mitochondrial disease according to the modified Walker criteria [30] were analyzed. The majority of samples were collected during routine clinic visits (n = 87 in 58 patients). Seven samples were collected from hospitalized patients; three patients contributed samples from both clinic visits and hospital stays, while four patients had samples collected only during hospitalization. Except where specifically noted, the analyses below relate to mitochondrial patients seen during clinic visits in relatively good health.

**Table 1:** Patient demographics.

Diagnosis	Number of Subjects	Gender (M:F)	Age (years [range; mean±SD])
Leigh syndrome:	18	14∶4	0.5–26.4; 6.4±5.8
Unknown defect (n = 6)			
Surf-1 deficiency (n = 4)			
Complex I deficiency (n = 3)			
Combined ETC defects (n = 3)			
Complex V deficiency (n = 2)			
ETC disorders:	10	5∶5	1.6–27.8; 13.3±7.5
Complex I deficiency (n = 4)			
Complex I+III deficiency (n = 2)			
Complex I+IV deficiency (n = 1)			
Complex IV deficiency (n = 3)			
MELAS (m.3243G>C)	8	6∶2	2.1–46.2; 20.6±15.5
mtDNA deletion syndrome:	7	5∶2	1.6–50.3; 15.0±14.9
KSS (n = 4)			
PS (n = 3)			
mtDNA depletion sydrome:	8	2∶6	0.5–27.0; 12.3±8.9
POLG1 (n = 4)			
dGK (n = 1)			
TK2 (n = 1)			
RRM2b (n = 1)			
Unknown defect (n = 1)			
Miscellaneous disorders:	11	1∶10	1.5–43.5; 16.0±13.8
Friedreich ataxia (n = 3)			
Complex V deficiency (n = 2)			
PDH deficiency (n = 2)			
MLASA (n = 2)			
Coenzyme Q_10_ deficiency (n = 1)			
Mitochondrial myopathy (n = 1)			
Total	62	33∶29	0.5–50.3; 13.6±12.2

Demographics for all patients, combining those seen in routine clinic visits and those hospitalized for metabolic crises, are shown. Abbreviations: dGK = deoxyguanosine kinase deficiency; FA = Friedreich ataxia; KSS = Kearns-Sayre syndrome; MELAS = mitochondrial encephalomyopathy, lactic acidosis and stroke-like episodes; MLASA = mitochondrial myopathy, lactic acidosis and sideroblastic anemia; PDH = pyruvate dehydrogenase deficiency; POLG1 = polymerase γ deficiency; PS = Pearson syndrome; RRM2B = ribonucleotide reductase M2 B deficiency; SURF1 = surf-1 deficiency; TK2 = thymidine kinase deficiency.

### Control Samples

We previously reported the mean whole blood GSH concentration in a control population (n = 59) of 900 µM±141, and GSSG of 1.17 µM±0.43 [29]. Using these measurements, the GSH/GSSG ratio was calculated as 881±374; and redox potential as −260 mV±6.4 (Table 2).

**Table 2:** Comparison of Glutathione Redox Status by Mitochondrial Disease Category.

Mitochondrial Disease Category	GSH (µM)	P-Value*	GSSG (µM)	P-Value*	GSH/GSSG Ratio	P-Value*	Redox potential (mV)	P-Value*
Leigh syndrome (n = 15)	735±125	<0.0001**	2.40±2.36	0.065	646±541	0.0516	−250±11.2	0.0046**
ETC disorders (n = 10)	847±112	0.2648	2.53±2.29	0.0935	626±618	0.2324	−251±11.3	0.0447**
MELAS (n = 8)	829±126	0.1807	1.66±0.82	0.1417	658±187	0.0149**	−255±4.2	0.0304**
mtDNA deletions (n = 7)	885±208	0.7999	2.53±0.85	0.0052**	453±249	0.0045**	−249±10.8	0.0420**
mtDNA depletion (n = 7)	870+190	0.6113	1.72±0.51	0.0032**	614±354	0.0773	−254±8.9	0.0249**
Miscellaneous (n = 11)	758+115	0.0025**	2.23±2.27	0.1550	572±345	0.0132**	−250±10.9	0.0091**
Metabolic Crisis (n = 7)	550±93	<0.0001**	1.76±1.00	0.5052	390±178	0.0306**	−242±7.2	0.0259**
Combined (n = 58)	808±149	0.0008**	2.23±1.84	<0.0001**	596±424	0.0002**	−251±9.7	<0.0001**
Controls (n = 59)	900±141		1.17±0.43		881±374		−260±6.4	

Glutathione indices for all mitochondrial disease patients combined, as well as for different subgroups of mitochondrial disease, are shown. The combined category excludes samples collected during times of metabolic crisis.

*comparing mitochondrial disease category to control, except for Metabolic Crisis category in which comparisons were made to mitochondrial disease patients not in crisis;

**significant at P<0.05.

### Mitochondrial Disease Samples

**Figure 1:**
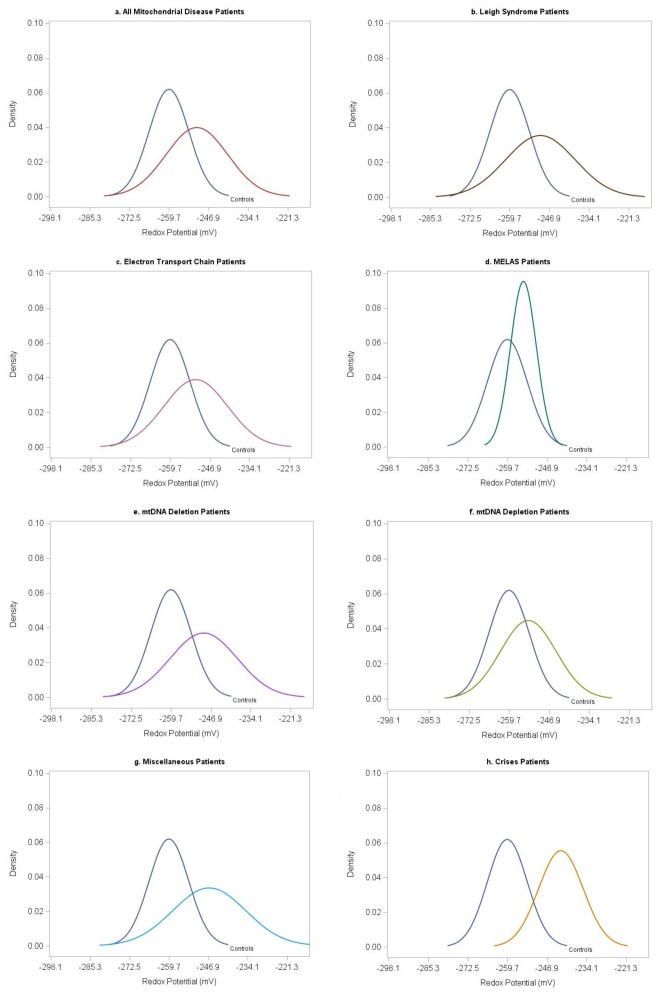
Glutathione redox potential distribution curves. The redox potential distribution curve for each mitochondrial disease subgroup is compared to the normal distribution of control redox potential in order to show how distribution of redox potential differs from the controls. a) Mitochondrial disease patients combined, excluding those in metabolic crisis; b) Leigh syndrome patients; c) Electron transport chain disorder patients; d) Mitochondrial encephalomyopathy, lactic acidosis and stroke-like episode patients; e) mtDNA deletion patients; f) mtDNA depletion patients; g) Miscellaneous mitochondrial disease patients; g) Mitochondrial disease patients hospitalized in metabolic crisis. Normal distribution plots were created with SAS 9.4. The density of the normal distribution is the height for a given value on the x-axis.

When all samples (n = 87) from clinically stable mitochondrial disease patients (n = 58) were analyzed in aggregate, results showed a significantly more oxidized redox potential (p<0.0001) than controls, as well as lower GSH levels (p = 0.0008) and GSH/GSSG ratio (p = 0.0002) and higher levels of GSSG (p<0.0001) (Table 2, Figure 1). These differences were not explained by potential differences in hematocrit or hemoglobin concentration, which were determined in 47 samples at the same time as glutathione collection. Six samples were associated with hemoglobin concentration <10 g/dL and/or hematocrit <30. There was no correlation between GSH concentrations or redox potential between samples obtained from these anemic patients compared to those with normal hemoglobin and hematocrit values (n = 41). Among all patients with mitochondrial disease, no significant differences in redox potential or glutathione levels were found between males and females, or between patients taking antioxidant supplements (n = 34) versus those who were not (n = 24). Patients taking antioxidants had a lower mean GSH level compared to those not taking antioxidant supplements (754 µM±139 v. 885 µM±128; p = 0.0006).

### Leigh Syndrome

Fifteen subjects with clinical and radiographic features consistent with Leigh syndrome were studied, including patients with a deficiency of complex I (n = 2), complex IV due to Surf1 deficiency (n = 3), complex V (n = 2), multiple ETC complexes (n = 3), or an unspecified biochemical abnormality (n = 5). A total of 17 blood samples collected during outpatient visits were analyzed (Table S1). The mean redox potential among Leigh syndrome patients was −250 mV±11.2, significantly more oxidized than in controls (p = 0.0046). In addition, whole blood GSH concentration in Leigh syndrome patients was significantly lower than controls (p<0.0001), as well as patients with ETC disorders without Leigh syndrome (p = 0.0218) and patients with mtDNA deletions (p = 0.0472) (Table 2). Glutathione levels and redox potential between Leigh syndrome subgroups were also compared in order to ascertain whether differences could be detected on the basis of type of underlying ETC complex deficiency, and no significant differences were found.

### ETC Disorders

Thirteen samples from 10 subjects with abnormalities of the mitochondrial ETC confirmed by enzymatic or molecular analyses were studied, including patients with an isolated deficiency of complex I (n = 4) or complex IV (n = 3), and those with combined deficiencies of complex I+III (n = 2) or complex I+IV (n = 1) (Table S2). The mean redox potential among all patients in this group (−251±11.3) was significantly more oxidized (p = 0.0447), although taken alone neither the GSH level nor GSSG level nor GSH/GSSG ratio achieved significance (Table 2). Because RONS production is especially associated with dysfunction of complexes I and III [11]–[14], data were also analyzed excluding the three subjects who did not have a deficiency of either of these complexes. No significant differences in redox potential, GSH, GSSG, or GSH/GSSG ratio were detected in this subset of mitochondrial disease patients (n = 7) compared to controls.

### MELAS

All MELAS patients studied (n = 8) had the common A to G transition at mitochondrial nucleotide position 3243 (m.3243A>G). Mutant load in blood was determined for six of the subjects and ranged from 12–70%. A total of 16 blood samples from MELAS patients were tested, with a mean GSH redox potential of −255±4.2 (p = 0.0304). GSH and GSSG levels were not significantly different than controls, while the GSH/GSSG ratio did achieve significance (p = 0.0149) (Table 2). Among the six MELAS patients for whom blood mutant load was known (Table S3), there was no correlation between this and any of the studied glutathione indices.

### mtDNA Deletions

A total of eleven blood samples from seven subjects with mtDNA deletion syndrome were analyzed. All patients had a large mtDNA deletion typically associated with Kearns-Sayre syndrome or Pearson syndrome, as well as consistent clinical features (Table S4). As a group, mtDNA deletion patients had a significantly more oxidized redox potential (p = 0.0420) and lower GSG/GSSG ratio (p = 0.0045) than controls, as well as higher levels of GSSG (p = 0.0052). GSH levels were only slightly lower than, but not statically different from, control values (Table 2). Blood mutant load was quantified in only two mtDNA deletion patients, so we were unable to perform further correlation analysis in this group. In addition, patients with either a Kearns-Sayre syndrome (n = 4) or Pearson syndrome (n = 3) phenotype were compared to each other and no significant differences in redox parameters were detected.

### mtDNA Depletion Syndrome

Blood samples (n = 15) from subjects (n = 7) with disorders causing mtDNA depletion were studied, including three patients with polymerase-γ deficiency, and one each with a deficiency of deoxyguanosine kinase, thymidine kinase 2 and ribonucleotide reductase M2B. The enzymatic diagnosis for one additional patient was unknown (Table S5). As a group, mtDNA depletion patients showed significantly oxidized redox potential (p = 0.0249) and higher GSSG levels (p = 0.0032) compared to controls, but GSH levels and GSH/GSSG were not significantly different (Table 2).

### Miscellaneous Mitochondrial Disorders

An additional 15 samples from subjects (n = 11) with mitochondrial disorders that did not fit into the above categories were also analyzed. This group included three patients with Friedreich ataxia, two with complex V deficiency, two with mitochondrial myopathy and sideroblastic anemia (MLASA), two with pyruvate dehydrogenase deficiency, one with coenzyme Q_10_ deficiency, and one with an unspecified mitochondrial myopathy (Table S6). Among these patients, redox potential was significantly more oxidized than controls (p = 0.0091), and GSH levels (p = 0.0025) and GSH/GSSG ratio (p = 0.0132) were significantly lower. No difference was noted in GSSG levels (Table 2).

### Clinical Status and NPMDS Scores

Seven samples were obtained from mitochondrial patients who were admitted to the hospital in metabolic crisis (Table S7). Among these patients, the mean redox potential was −242 mV±7.2, which was significantly more oxidized than all mitochondrial patients who were in relatively good health (p = 0.0259). The mean concentrations of whole blood GSH (550 µM±93) and GSH/GSSG ratio (390±178) were significantly lower than controls (p<0.0001 and p = 0.0306 respectively), while the mean GSSG concentration (1.76 µM±1.00) was not significantly different from controls. NPMDS scores were evaluated for 29 patients (Tables S1 to S7), and were analyzed as the combined score from sections I to III (i.e., an overall objective clinical severity score), quality of life score (section IV) and total score (sections I to IV combined). Although there was a general trend of increasing NPMDS score with a more oxidized redox potential, a significant correlation was not observed between NPMDS score and any of the glutathione indices.

## Discussion

Using a sensitive LC-MS/MS assay for measuring whole blood glutathione, we have demonstrated significant redox imbalance in a large cohort of patients with mitochondrial disease, with the greatest abnormality seen in patients who were acutely ill. This study represents the largest single collection of mitochondrial patients who have had redox status examined to date, and allowed for the further subdivision of patients by disease subgroup based on molecular etiology and/or clinical category. In addition, our robust analytical approach, consistently applied to all samples analyzed, was validated in the clinical setting and specifically controlled for artifacts of sample collection and handling including the autooxidation of GSH to GSSG [29]. This study extends our previous report of GSH deficiency in leukocytes from mitochondrial disease and organic acidemia patients as analyzed by Hi-D FACS [28], a semi-quantitative technique that measures intracellular GSH but not GSSG, and is not wholly amenable to the clinical setting.

Precise determinations of whole blood reduced and oxidized glutathione allowed us to calculate the glutathione redox potential using the Nernst equation, which accounts for both the intrinsic properties of the redox couple as well as their relative concentrations [32]. Because the glutathione system plays a critical role maintaining overall redox status of the body [34], the GSSG/2GSH redox couple can be taken as representative of the redox environment of a biological system [33]. Although absolute levels of GSH and GSSG varied substantially within and between patients and provided only a limited picture of redox status, the calculated redox potential was a more stable indicator and provided additional insights. Only a limited number of patients have undergone serial sampling; however, it is interesting to note that the two patients who were evaluated on four or more occasions (patients 29 and 45) had relatively stable redox potentials. Several patients (e.g. patients 3, 31, 34, 37 and 41) showed a moderate degree of variability in redox potential during the course of the study. In some instances, fluctuation was associated with changes in co-factor supplementation. Further longitudinal studies on a larger cohort are needed in order to determine both the degree and significance of any fluctuations in redox status in individual patients. Our calculated whole blood glutathione redox potentials are in good agreement with previously reported estimates of redox potential in tissues, which range between −260 mV and −185 mV [35], [36]. Using this approach, we demonstrate that redox potential in mitochondrial disease patients shows significant redox imbalance, with an increased level of oxidation of approximately 9 mV over controls. Moreover, mitochondrial disease patients who were hospitalized for treatment of an acute metabolic crisis had lower GSH levels and more pronounced redox imbalance. In the hospitalized patients, other co-morbid conditions, such as intercurrent viral illness, exacerbation of seizures, hyperglycemia, or metabolic acidosis, may have contributed to the baseline mitochondrial stress inherent in these disorders. In addition, although further studies on a larger number of hospitalized patients are needed, our preliminary findings suggest that the severity of the co-morbid condition may also play a role in determining ultimate redox status. For example, one patient in status epilepticus had a redox potential of −232 mV compared to a potential of −247 mV in another patient admitted with seizure exacerbation who was not in status epilepticus at the time of blood sampling (Table S7). In short, mitochondrial disease patients as a whole appear to have a more oxidized redox status at baseline compared to controls, and redox status becomes more oxidized in times of crisis.

In contrast to previous studies combining mitochondrial patients into a single, heterogeneous group [28], our population size allowed us to classify patients into subgroups based on phenotype and biochemical or molecular pathology (Tables S1 to S7) in order to evaluate whether redox status varies depending on the underlying phenotype, biochemical defect, or both. While abnormalities in glutathione levels and redox potential were observed in all subgroups of mitochondrial disease, our results indicate that there may be differences in levels of GSH, GSSG and the redox potential even between subgroups (Table 2). Interestingly, whole blood GSH levels were significantly lower in Leigh syndrome and miscellaneous categories of mitochondrial disease, but not in other subgroups.

Although we were able to classify patients into subgroups, considerable heterogeneity related to underlying molecular and biochemical features was still present within each category of mitochondrial disease. For example, the Leigh syndrome cohort, although sharing a classic mitochondrial disease clinical phenotype, was characterized by biochemical or molecular defects that affected a variety of respiratory chain subunits. Nevertheless, as a group, the Leigh syndrome patients demonstrated clear redox abnormalities. Interestingly, a recent open-label study using EPI-743, a novel redox-modulating drug, to treat a heterogeneous group of 10 Leigh syndrome patients demonstrated clinical improvement in all patients, despite the presence of multiple underlying molecular etiologies [37]. Such a therapeutic response across multiple genetic causes suggests that a common pathway related to pathogenesis may exist in Leigh syndrome. Studies of additional patients are needed in order to more clearly elucidate differences between mitochondrial subgroups and the role of oxidative stress in the pathophysiology of mitochondrial dysfunction caused by different biochemical or molecular defects.

The relatively normal levels of whole blood GSH observed in patients with pathological mtDNA mutations or deletions may in part be due to *in vivo* selection. In tissues with a high turnover, such as the hematopoietic system, stem cells with relatively normal mtDNA content have a selective advantage and, subsequently, mutant load in blood may be low [38], [39]. Heteroplasmy data were available for seven MELAS patients, and only two had >50% mutant load in blood (Table S3). Although all MELAS patients had evidence of neurological impairment, the peripheral blood mutant load may not have reflected the degree of heteroplasmy present in other parts of the body. Given these considerations, it is not surprising that we did not identify a direct relationship between blood mutant load and redox status in MELAS. A recent study in 14 Japanese MELAS patients detected evidence for peripheral redox imbalance by measuring diacron-reactive oxygen metabolites and biological antioxidant potential testing. Although mutant load was not reported, the patients studied had more severe clinical symptoms than our cohort [40]. Further studies in MELAS patients with more significant clinical disease and higher levels of mutant load in peripheral blood would be needed in order to study the potential relationship between mutant load and redox status in more detail. Only two of our mtDNA deletion patients had blood heteroplasmy analysis, so it was not possible to study the relationship between blood mutant load and redox status in this group.

The demonstration that redox abnormalities are present in mitochondrial disease patients even when relatively well, and further worsen during periods of acute illness (Table S7), is consistent with reports correlating changes in the half-cell reduction potential of the GSSG/2GSH with the biological status of the cell. These reports note the progression from proliferation to differentiation and, finally, cell death being associated with an increasingly oxidized redox state [33]. The degree of oxidation is related to the likelihood of cells undergoing apoptosis or necrosis; a moderate, but lethal oxidative stimulus causes apoptosis, whereas a severe stimulus results in necrosis [33], [41]–[43]. This response may be mediated by redox-dependent interactions with downstream signaling pathways involving release of apoptogenic factors including cytochrome *c* and apoptosis inducing factor [44]–[46]. Decreased cellular GSH precedes the release of cytochrome *c* and, therefore, redox status may represent the major determinant of the apoptotic switch [33], [43]. Redox status has also been closely linked to a number of other intracellular signaling cascades via redox-sensitive transcription factors, including activator protein-1, nuclear factor kappa B, NE-F2 related factor, and p53 [47]–[50]. Therefore, *in vivo* GSH deficiency and a relatively oxidized redox status in individuals with mitochondrial disorders are likely to be associated with significant perturbation of these signaling pathways, an observation which could potentially inform the development of new therapeutic approaches for these disorders.

Our earlier work using Hi-D FACS to study white blood cell subsets from patients with primary or secondary mitochondrial dysfunction showed that antioxidant supplements improved iGSH levels [28]. In contrast, mean whole blood GSH level was lower in patients taking antioxidants compared to those not taking supplements during the time of sample collection, and no significant differences were noted in GSSG, GSH/GSSG ratio or redox potential between these two groups. The lack of uniformity of antioxidant supplementation with respect to types of supplements used and dosing makes comparisons between those taking or not taking supplements problematic. Patients taking antioxidants may also have been doing so because of the presence of more severe disease, which could potentially explain the lower mean GSH level. Given the particularly low GSH levels in Leigh syndrome patients, it seems likely that disease severity among studied cohorts has played some role in these findings. The earlier study of 21 mitochondrial disease patients included only a single Leigh syndrome patient, while the majority had various ETC disorders or MELAS [28]. An open-label clinical trial using EPI-743, a novel *para*-benzoquinone analog with electron cycling capacity, in 10 Leigh syndrome patients who had a variety of underlying molecular or enzymatic defects showed a significant increase in GSH levels in lymphocytes following the start of therapy [21]. Similar to the whole blood findings reported in the current study, Pastore *et al*. detected a significant decrease in leukocyte GSH and increased oxidized forms of glutathione in the Leigh syndrome patients at baseline [21]. Therefore, it is also possible that white blood cells are more sensitive to changes related to antioxidant therapy, but further studies directly comparing measurement of redox status in different blood compartments in patients who have mitochondrial function caused by a variety of etiologies are needed to clarify this point.

In summary, we report significant differences in glutathione status and redox potential in patients with mitochondrial disorders compared to controls. These abnormalities were present even during times of relative health, but were exacerbated in times of metabolic crisis. The greatest degree of oxidation during periods of relative health was observed in patients with the most severe clinical manifestation of mitochondrial disease including Leigh syndrome, while even more severe redox imbalance was present in patients in the midst of a metabolic crisis. Although our findings are preliminary, the measurement of redox potential via the GSSG/2GSH couple holds promise as a biomarker for mitochondrial dysfunction, and may also yield further insights related to variation of oxidative metabolism between different subtypes of mitochondrial disease.

## Supporting Information

Table S1 **Leigh syndrome patients.**
(DOC)

Table S2 **Electron transport chain abnormality patients.**
(DOC)

Table S3 **Mitochondrial encephalomyopathy, lactic acidosis and stroke-like episodes (MELAS) patients.**
(DOC)

Table S4 **mtDNA deletion syndrome patients.**
(DOC)

Table S5 **mtDNA depletion syndrome patients.**
(DOC)

Table S6 **Miscellaneous mitochondrial disorders patients.**
(DOC)

Table S7 **Mitochondrial patients hospitalized for “metabolic crisis”.**
(DOC)
