# Supplementary material for: Degree of Glutathione Deficiency and Redox Imbalance Depend on Subtype of Mitochondrial Disease and Clinical Status
Source: PLoS One. 2014 Jun 18;9(6):e100001. doi: 10.1371/journal.pone.0100001 (PMC4062483; doi:10.1371/journal.pone.0100001)
Supplement: Table S1 — Leigh syndrome patients. (DOC) [file pone.0100001.s001.doc]

| Patient/ Gender | Age (years) | Diagnosis^1^ | GSH (uM) | GSSG (uM) | GSH/  GSSG | Redox potential (mV) | Other  supplements^2^ | Newcastle scores^3^ |
| --- | --- | --- | --- | --- | --- | --- | --- | --- |
| 1/M | 6.7 | Leigh syndrome (Complex I, m.10158T>C) | 753 | 0.87 | 866 | -250 | Carnitine, arginine, creatine, B2, folinic acid, Q | 28/11.7/39.7 |
| 2/M | 7.8 | Leigh syndrome (Complex I) | 665 | 0.42 | 1583 | -265 | Carnitine, BC | 19/12.1/31.1 |
| 3/F | 1.9 | Leigh syndrome (Complex I+III, II, IV) | 940 | 14.54 | 65 | -228 | Carnitine, biotin, B1, B2, C, E, Q |  |
|  | 7.1 |  | 601 | 2.61 | 230 | -238 | “ |  |
|  | 8.1 |  | 512 | 0.79 | 648 | -250 | “ |  |
| 4/F | 4.2 | Leigh syndrome (Complex II+III, IV) | 742 | 0.36 | 2061 | -270 | Carnitine, B2, B6, LA, melatonin | 27/17.1/44.1 |
| 5/M | 6.7 | Leigh syndrome (Complex I, IV) | 654 | 1.28 | 510 | -250 | Carnitine, BC, Q | 39/15.4/54.4 |
| 6/M | 2.4 | Leigh syndrome (Surf1 deficiency) | 779 | 2.34 | 333 | -246 | Carnitine, Q | 28/17.1/45.1 |
| 7/M | 5.6 | Leigh syndrome (Surf1 deficiency) | 523 | 0.6 | 871 | -254 | Carnitine, BC, C, E, LA, Q | 24/7.5/31.5 |
| 8/M | 26.4 | Leigh syndrome (Surf1 deficiency) | 1036 | 2.93 | 354 | -251 | Carnitine, folinic acid |  |
| 9/M | 2.4 | Leigh syndrome (Complex V) | 620 | 2.7 | 230 | -239 | Carnitine, Q | 14/10.8/24.8 |
| 10/M | 8.9 | Leigh syndrome (Complex V) | 713 | 0.94 | 759 | -256 | Carnitine, biotin, B6, C, D, E, Q | 20/12.5/32.5 |
| 11/M | 13.7 | Leigh syndrome (unspecified) | 770 | 1.3 | 592 | -254 | None |  |
| 12/F | 2.7 | Leigh syndrome (unspecified) | 691 | 9.1 | 76 | -226 | Carnitine, B2, folic acid, Q, melatonin | 31/15/46 |
| 13/M | 10.9 | Leigh syndrome (unspecified) | 956 | 1.61 | 593 | -257 | Carnitine | 33/5/38 |
| 14/M | 2.3 | Leigh syndrome (unspecified) | 687 | 2.14 | 321 | -244 | Carnitine, BC, Q |  |
| 15/M | 3.8 | Leigh syndrome (unspecified) | 748 | 3.39 | 221 | -241 | None |  |

^1^Electron transport chain deficiency and/or molecular defect are shown where known; ^2^Abbreviations: B1=thiamine; B2=riboflavin; B6=pyridoxine; BC=vitamin B complex; C=vitamin C; D=vitamin D; E=vitamin E; LA=α-lipoic acid; Q=coenzyme Q_10_; ^3^Newcastle Paediatric Mitochondrial Disease Scale (NPMDS) scores are shown for sections I to III combined/section IV/sections I to IV combined.
