# Supplementary material for: Degree of Glutathione Deficiency and Redox Imbalance Depend on Subtype of Mitochondrial Disease and Clinical Status
Source: PLoS One. 2014 Jun 18;9(6):e100001. doi: 10.1371/journal.pone.0100001 (PMC4062483; doi:10.1371/journal.pone.0100001)
Supplement: Table S2 — Electron transport chain abnormality patients. (DOC) [file pone.0100001.s002.doc]

| Patient/ Gender | Age (years) | Diagnosis^1^ | GSH (uM) | GSSG (uM) | GSH/GSSG | Redox potential (mV) | Other supplements^2^ | Newcastle scores^3^ |
| --- | --- | --- | --- | --- | --- | --- | --- | --- |
| 16/M | 16.8 | Complex I deficiency (m.14459G>A) | 979 | 0.43 | 2277 | -274 | None |  |
| 17/F | 9.7 | Complex I deficiency (m.14459G>A) | 1003 | 1.46 | 687 | -259 | None |  |
| 18/F | 15.0 | Complex I deficiency (m.13513G>A) | 841 | 1.86 | 452 | -251 | C |  |
|  | 15.9 |  | 876 | 1.86 | 472 | -252 | BC, MitoTonic |  |
| 19/F | 18.3 | Complex I deficiency (m.13513G>A) | 842 | 4.21 | 200 | -241 | Q |  |
|  | 18.6 |  | 696 | 1.59 | 437 | -249 | Arginine, BC, Q |  |
|  | 18.9 |  | 713 | 0.94 | 759 | -256 | “ |  |
| 20/F | 27.8 | Complex I +III deficiency | 836 | 8.47 | 99 | -232 | D, LA, Q | 42/74.3 |
| 21/M | 7.2 | Complex I+III deficiency | 755 | 1.94 | 389 | -248 | Carnitine, arginine, folic acid, B12, K, Q | 12/8.1/20.1 |
| 22/M | 4.6 | Complex I+IV deficiency | 778 | 2.78 | 280 | -244 | Carnitine | 42/16.7/58.7 |
| 23/F | 14.6 | Complex IV deficiency | 1021 | 3.77 | 271 | -247 | None |  |
| 24/M | 1.6 | Complex IV deficiency | 660 | 1.39 | 475 | -249 | Carnitine, BC, Q |  |
| 25/M | 3.7 | Complex IV deficiency (COX10 deficiency) | 825 | 0.97 | 851 | -259 | B1, LA, Q | 34/15.4/49.4 |

^1^Electron transport chain deficiency and/or molecular defect are shown where known; ^2^Abbreviations: B1=thiamine; B12=vitamin B_12_; BC=vitamin B complex; C=vitamin C; D=vitamin D; LA=α-lipoic acid; Q=coenzyme Q_10_; ^3^Newcastle Paediatric Mitochondrial Disease Scale (NPMDS) scores are shown for sections I to III combined/section IV/sections I to IV combined. For patient 20, the Newcastle Mitochondrial Disease Adult Scale (NMDAS) was used and scores are shown for sections I to III combined/quality of life (SF-12v2 Health Survey).
