# Supplementary material for: Degree of Glutathione Deficiency and Redox Imbalance Depend on Subtype of Mitochondrial Disease and Clinical Status
Source: PLoS One. 2014 Jun 18;9(6):e100001. doi: 10.1371/journal.pone.0100001 (PMC4062483; doi:10.1371/journal.pone.0100001)
Supplement: Table S3 — Mitochondrial encephalomyopathy, lactic acidosis and stroke-like episodes (MELAS) patients. (DOC) [file pone.0100001.s003.doc]

| Patient/ Gender | Age (years) | Clinical Features^1^ | **Mutant load^2^** | GSH (uM) | GSSG (uM) | GSH/  GSSG | Redox potential (mV) | Others  supplements^3^ | Newcastle scores^4^ |
| --- | --- | --- | --- | --- | --- | --- | --- | --- | --- |
| 26/M | 3.9 | Encephalopathy, dystonia, weakness, died at age 9 years | 12% | 896 | 1.34 | 669 | -257 | None |  |
|  | 7.9 |  |  | 1033 | 1.63 | 634 | -258 | Carnitine, B1, B2, B5, C, E, Q |  |
|  | 8.6 |  |  | 911 | 1.80 | 506 | -254 | “ | 44/18/62.8 |
| 27/M | 16.5 | Seizures, stroke-like episodes, weakness, sensorineural deafness, episodic ataxia and confusion, died at age 19 years | 70% | 705 | 0.85 | 829 | -257 | None |  |
| 28/M | 39.8 | Episodic facial numbness, nerve pain, insulin-dependent diabetes | 66% | 946 | 1.17 | 809 | -260 | None |  |
| 29/M | 25.5 | Seizures, severe cognitive impairment, stroke-like episodes, died at age 30 years | 40% | 895 | 0.79 | 1132 | -264 | Carnitine, arginine, BC, B2, biotin, K, Q |  |
|  | 26.2 |  |  | 744 | 1.02 | 729 | -256 | “ |  |
|  | 26.9 |  |  | 482 | 0.55 | 876 | -253 | “ |  |
|  | 27.5 |  |  | 708 | 0.78 | 908 | -258 | None | 30/72.7 |
| 30/F | 2.1 | Severe hypotonia, developmental delay, ptosis, failure to thrive | ND | 1000 | 2.13 | 469 | -254 | Carnitine, C, E, LA, Q |  |
|  | 2.8 |  |  | 857 | 0.89 | 963 | -261 | “ |  |
|  | 4.5 |  |  | 1015 | 4.6 | 220 | -245 | “ |  |
| 31/M | 45.2 | Seizures, stroke-like episodes, severe cognitive impairment, sensorineural hearing loss, insulin-dependent diabetes | 12% | 845 | 4.42 | 191 | -240 | Arginine, folinic acid, Q |  |
|  | 46.2 |  |  | 870 | 0.82 | 1061 | -263 | “ |  |
| 32/M | 17.7 | Seizures, vision abnormalities, sensorineural hearing loss, progressive dementia, leukoencephalopathy | ND | 642 | 1.06 | 606 | -252 | B1, Q |  |
| 33/F | 40.8 | Myoclonic jerks, corneal dystrophy, intermittent right-sided numbness and weakness, failure to gain weight, memory problems | 34% | 868 | 2.65 | 328 | -248 | Q |  |

^1^All patients had significant clinical involvement and symptoms associated with the MELAS spectrum phenotype; ^2^All patients carried the common m.3243A>G mutation. Mutant load in blood is shown where known. ND=not done; ^3^Abbreviations: B1=thiamine; B2=riboflavin; B5=pathothenic acid; BC=vitamin B complex; C=vitamin C; E=vitamin E; K=vitamin K; LA=α-lipoic acid; Q=coenzyme Q_10_; ^4^Newcastle Paediatric Mitochondrial Disease Scale (NPMDS) scores are shown for sections I to III combined/section IV/sections I to IV combined. For patient 29, the Newcastle Mitochondrial Disease Adult Scale (NMDAS) was used and scores are shown for sections I to III combined/quality of life (SF-12v2 Health Survey).
