# Supplementary material for: Degree of Glutathione Deficiency and Redox Imbalance Depend on Subtype of Mitochondrial Disease and Clinical Status
Source: PLoS One. 2014 Jun 18;9(6):e100001. doi: 10.1371/journal.pone.0100001 (PMC4062483; doi:10.1371/journal.pone.0100001)
Supplement: Table S4 — mtDNA deletion syndrome patients. (DOC) [file pone.0100001.s004.doc]

| Patient/ Gender | Age (years) | Diagnosis^1^ | GSH (uM) | GSSG (uM) | GSH/  GSSG | Redox potential (mV) | Other medications^2^ | Newcastle scores^3^ |
| --- | --- | --- | --- | --- | --- | --- | --- | --- |
| 34/F | 44.7 | Kearns–Sayre syndrome | 1228 | 0.85 | 1445 | -271 | None |  |
|  | 50.3 |  | 1079 | 2.58 | 418 | -254 | Carnitine, BC, C, LA, Q |  |
| 35/M | 11.0 | Kearns–Sayre syndrome | 1001 | 2.46 | 407 | -252 | None | 44/19.6/63.6 |
| 36/M | 17.7 | Kearns–Sayre syndrome | 882 | 1.59 | 555 | -255 | Carntine, E, folinic acid, Q |  |
|  | 19.8 |  | 1007 | 1.85 | 544 | -256 | “ |  |
| 37/M | 13.5 | Kearns–Sayre syndrome | 686 | 1.21 | 567 | -252 | Carnitine, BC, Q |  |
|  | 14.0 |  | 785 | 4.01 | 196 | -240 | “ | 42/9.2/51.2 |
| 38/M | 3.1 | Pearson syndrome^4^ | 525 | 4.25 | 124 | -228 | NAC |  |
| 39/F | 1.6 | Pearson syndrome^4^ | 826 | 2.57 | 322 | -247 | None |  |
| 40/M | 3.7 | Pearson syndrome^4^ | 1099 | 3.11 | 353 | -252 | Folinic acid |  |
|  | 4.6 |  | 915 | 1.62 | 563 | -255 | None | 19/15.8/34.8 |

^1^All patients carry a large mtDNA deletion; ^2^Abbreviations: BC=vitamin B complex; C=vitamin C; E=vitamin E; LA=α-lipoic acid; NAC=*N-*acetylcysteine; Q=coenzyme Q_10_; ^3^Newcastle Paediatric Mitochondrial Disease Scale (NPMDS) scores are shown for sections I to III combined/section IV/sections I to IV combined; ^4^The Pearson syndrome patients were stable from a hemodynamic standpoint at the time of blood sampling and did not undergo blood transfusion or have clinically significant anemia during the period of study: Patient 38 hemoglobin=11.8 g/dL and hematocrit=35.2%; Patient 39 hemoglobin=9.9 g/dL and hematocrit=29.4; Patient 40 hemoglobin=9.9 g/dL and hematocrit=29.3% for the 1^st^ sample and hemoglobin=10.2 g/dL and hematocrit=29.5% for the 2^nd^ sample.
