# Supplementary material for: Degree of Glutathione Deficiency and Redox Imbalance Depend on Subtype of Mitochondrial Disease and Clinical Status
Source: PLoS One. 2014 Jun 18;9(6):e100001. doi: 10.1371/journal.pone.0100001 (PMC4062483; doi:10.1371/journal.pone.0100001)
Supplement: Table S5 — mtDNA depletion syndrome patients. (DOC) [file pone.0100001.s005.doc]

| Patient/ Gender | Age (years) | Diagnosis^1^ | GSH (uM) | GSSG (uM) | GSH/  GSSG | Redox potential (mV) | Other supplements^2^ | Newcastle scores^3^ |
| --- | --- | --- | --- | --- | --- | --- | --- | --- |
| 41/F | 9.4 | Polymerase γ deficiency | 1121 | 1.32 | 849 | -263 | Carnitine, BC, B12, C, E, LA, Q |  |
|  | 12.2 |  | 1183 | 0.60 | 1972 | -275 | “ |  |
|  | 13.3 |  | 703 | 0.60 | 1171 | -261 | “ | 28/9.8/37.8 |
| 42/F | 4.3 | Polymerase γ deficiency | 763 | 0.89 | 857 | -258 | None | 40/17.1/57.1 |
| 43/F | 8.8 | Polymerase γ deficiency | 862 | 1.54 | 559 | -255 | Carnitine, arginine, melatonin |  |
| 44/M | 1.3 | Deoxyguanosine kinase deficiency | 966 | 1.97 | 490 | -254 | Carnitine, BC, C, E, Q |  |
|  | 6.7 |  | 818 | 2.11 | 387 | -249 | “ |  |
|  | 7.5 |  | 922 | 2.01 | 460 | -253 | “ |  |
| 45/F | 22.5 | Thymidine kinase 2 deficiency | 961 | 1.48 | 649 | -258 | Carnitine, BC, C |  |
|  | 22.5 |  | 1035 | 1.97 | 525 | -256 | “ |  |
|  | 22.5 |  | 1091 | 0.93 | 1173 | -267 | “ |  |
|  | 25.9 |  | 1163 | 1.74 | 668 | -261 | “ |  |
|  | 27.0 |  | 778 | 2.50 | 311 | -246 | None |  |
| 46/F | 0.5 | Ribonucleotide reductase M2 B deficiency | 715 | 1.68 | 426 | -248 | Carnitine, BC, B12, C, E, LA | 43/21.9/64.9 |
| 47/M | 5.2 | mtDNA depletion, unspecified | 602 | 2.08 | 289 | -241 | Carnitine, B2, D, LA, Q | 36/12.5/48.5 |

^1^Defects associated with mtDNA depletion are shown where known; ^2^Abbreviations: B2=riboflavin; B12=vitamin B_12_; BC=vitamin B complex; C=vitamin C; D=vitamin D; E=vitamin E; LA=α-lipoic acid; Q=coenzyme Q_10_; ^3^Newcastle Paediatric Mitochondrial Disease Scale (NPMDS) scores are shown for sections I to III combined/section IV/sections I to IV combined.
