# Supplementary material for: Degree of Glutathione Deficiency and Redox Imbalance Depend on Subtype of Mitochondrial Disease and Clinical Status
Source: PLoS One. 2014 Jun 18;9(6):e100001. doi: 10.1371/journal.pone.0100001 (PMC4062483; doi:10.1371/journal.pone.0100001)
Supplement: Table S6 — Miscellaneous mitochondrial disorders patients. (DOC) [file pone.0100001.s006.doc]

| Patient/ Gender | Age (years) | Diagnosis^1^ | GSH (uM) | GSSG (uM) | GSH/  GSSG | Redox potential (mV) | Other supplements^2^ | Newcastle scores^3^ |
| --- | --- | --- | --- | --- | --- | --- | --- | --- |
| 48/F | 9.2 | Complex V deficiency (m.9056C>T) | 938 | 2.57 | 365 | -250 | B6 |  |
| 49/M | 12.2 | Complex V  (m.8993T>G, NARP) | 826 | 1.43 | 577 | -254 | BC | 17/13.8/30.8 |
| 50/F | 24.2 | MLASA | 564 | 1.62 | 349 | -243 | Q |  |
|  | 27.3 |  | 636 | 0.52 | 1224 | -261 | C, Q |  |
| 51/F | 40.4 | MLASA | 641 | 10.23 | 63 | -222 | B6, B12, D, Q |  |
|  | 43.5 |  | 732 | 7.22 | 101 | -230 | Creatine, BC, B6, B12, D, E, Q |  |
| 52/F | 15.0 | Coenzyme Q_10_ deficiency | 777 | 2.89 | 269 | -244 | B1, C, E, Q |  |
| 53/F | 3.7 | Mitochondrial myopathy | 683 | 1.31 | 521 | -251 | Carnitine, biotin, B1, B2, B5, C, E LA, Q |  |
| 54/F | 10.6 | Friedreich ataxia | 882 | 0.65 | 1357 | -266 | BC, C, D, E, Selinium |  |
| 55/F | 16.9 | Friedreich ataxia | 854 | 1.75 | 488 | -253 | E | 40/10.5/50.5 |
| 56/F | 33.6 | Friedreich ataxia | 713 | 0.8 | 894 | -258 | None | 72/82.2 |
| 57/F | 5.5 | Pyruvate dehydrogenase deficiency | 658 | 1.6 | 410 | -247 | B1 |  |
|  | 6.3 |  | 501 | 0.66 | 759 | -251 | B1 |  |
| 58/F | 1.5 | Pyruvate dehydrogenase deficiency | 789 | 2.49 | 316 | -246 | B1, biotin |  |
|  | 2.0 |  | 807 | 1.90 | 426 | -250 | B1 |  |

^1^MLASA=mitochondrial myopathy, lactic acidosis and sideroblastic anemia; NARP=Neurogenic muscle weakness, ataxia and retinitis pitmentosa; ^2^Abbreviations: B1=thiamine; B2=riboflavin; B5=pantothenic acid; B6=pyridoxine; B12=vitamin B_12_; BC=vitamin B complex; C=vitamin C; D=vitamin D; E=vitamin E; LA=α-lipoic acid; Q=coenzyme Q_10_; ^3^Newcastle Paediatric Mitochondrial Disease Scale (NPMDS) scores are shown for sections I to III combined/section IV/sections I to IV combined. For patient 56, the Newcastle Mitochondrial Disease Adult Scale (NMDAS) was used and scores are shown for sections I to III combined/quality of life (SF-12v2 Health Survey).
