# Supplementary material for: Degree of Glutathione Deficiency and Redox Imbalance Depend on Subtype of Mitochondrial Disease and Clinical Status
Source: PLoS One. 2014 Jun 18;9(6):e100001. doi: 10.1371/journal.pone.0100001 (PMC4062483; doi:10.1371/journal.pone.0100001)
Supplement: Table S7 — Mitochondrial patients hospitalized for “metabolic crisis”. (DOC) [file pone.0100001.s007.doc]

| Patient/ Gender | Age (years) | Diagnosis^1^ | Co-morbid Conditions | GSH (uM) | GSSG (uM) | GSH/  GSSG | Redox potential (mV) | Other supplements^2^ | Newcastle scores^3^ |
| --- | --- | --- | --- | --- | --- | --- | --- | --- | --- |
| 59/M | 1.8 | Leigh syndrome (Complex I) | Seizures, respiratory failure requiring intubation and ventilation | 488 | 0.86 | 568 | -247 | B1, B2, Q | 41/21.9/62.9 |
| 60/M | 5.6 | Leigh syndrome (Surf1 deficiency) | Respiratory syncytial virus bronchiolitis, intermittent desaturations | 600 | 3.73 | 161 | -234 | NAC | 35/21.3/56.3 |
| 61/F | 0.5 | Leigh syndrome, unspecified | Developmental regression, respiratory distress | 494 | 0.82 | 602 | -248 | Carnitine, BC, Q |  |
| 62/F | 7.3 | Polymerase γ deficiency | Status epilepticus, respiratory failure requiring intubation and ventilation | 411 | 1.95 | 211 | -232 | Carntine, E, Q | 40/15.8/55.8 |
| 28/M | 8.8 | m.3243A>G (MELAS) | Choreoathetoid storm following upper respiratory infection, episodic emesis | 613 | 1.18 | 519 | -249 | None |  |
| 37/M | 11.4 | mtDNA deletion (Kearns-Sayre syndrome) | Asthma exacerbation, poor peripheral perfusion, lactic acidosis, hyperglycemia | 560 | 1.99 | 281 | -240 | Carnitine, BC, Q |  |
| 53/F | 3.6 | Mitochondrial myopathy | Upper respiratory infection, lactic acidosis, elevated transaminases and creatine kinase | 687 | 1.78 | 386 | -252 | Carnitine, biotin, B1, B2, B5, C, E LA, Q |  |

^1^Clinical phenotypes, electron transport chain deficiency and/or molecular defect are shown. MELAS=mitochondrial encephalomyopathy, lactic acidosis and stroke-like episodes; ^2^Abbreviations: B1=thiamine; B2=riboflavin; B5=pantothenic acid; BC=vitamin B complex; C=vitamin C; E=vitamin E; LA=α-lipoic acid; NAC=*N*-acetylcysteine; Q=coenzyme Q_10_; ^3^Newcastle Paediatric Mitochondrial Disease Scale (NPMDS) scores are shown for sections I to III combined/section IV/sections I to IV combined.
